# Prognostic values of GPNMB identified by mining TCGA database and STAD microenvironment

**DOI:** 10.18632/aging.103646

**Published:** 2020-08-21

**Authors:** Kunhou Yao, Lunshou Wei, Junjie Zhang, Chenyu Wang, Chaoyang Wang, Changjiang Qin, Song Li

**Affiliations:** 1 Henan University, Kaifeng 475000, Henan, P.R. China; 2 Department of General Surgery, Huaihe Hospital of Henan University, Kaifeng 475000, Henan, P.R. China; 3 Department of Urology, Huaihe Hospital of Henan University, Kaifeng 475000, Henan, P.R. China; 4 Department of Gastroenterology, Huaihe Hospital of Henan University, Kaifeng 475000, Henan, P.R. China

**Keywords:** microenvironment, differently expressed genes, hub genes, miRNA, GPNMB

## Abstract

The survival rate of stomach adenocarcinoma patients with immune and stromal scores and different clinicopathological features obtained from the TCGA datasets was systematically compared. A list of genes that are correlated with stomach adenocarcinoma microenvironment were extracted using the TCGA database to predict the prognosis and survival. In addition, the differentially expressed genes were extracted by comparing the immune and stromal scores of the groups. The protein-protein interaction network, and functional and pathway enrichment analyses of differentially expressed genes were performed. A total of 8 hub genes were selected from the differentially expressed genes to predict the overall survival and disease-free survival rates. GPNMB was selected from the hub genes based on the survival and prognosis analyses. A nomogram was built by including the potential risk factors based on multivariate Cox analysis. Cell function experiments and xenograft tumors were conducted *in vivo* to further verify the role of GPNMB in tumor progression. The predicted microRNA, miR-30b-3p, might act as upstream negative regulator and binding to 3’ UTR of GPNMB, confirming by fluorescent enzyme reporter gene experiment. In summary, immune-related scores are crucial factors in the malignant progression of stomach adenocarcinoma and GPNMB acts as a potentially useful prognostic factor for stratification and in developing the treatment strategy

## INTRODUCTION

Stomach adenocarcinoma (STAD) is one of the most common malignant tumors of the digestive tract. In 2018, there were approximately 1,033,701 new cases and approximately 782,685 deaths associated with STAD worldwide, and most of these were in the locally advanced stage at the time of diagnosis [1, 2]. The mechanism of occurrence and development of STAD is still unclear. The current treatments of STAD include surgery, radiation therapy, chemotherapy, etc. However, the incidence of local recurrence or distant metastasis of gastric cancer is 40% to 70% after surgery, and the side effects associated after undergoing radiotherapy and chemotherapy are obvious [3]. Therefore, the prevention and control of STAD has become an urgent public health issue, and it is necessary to explore the underlying mechanism of STAD progression in order to find new therapeutic and diagnostic targets that can improve the survival rate of patients.

With the concept of "survival with tumor", the tumor immune microenvironment has attracted much attention and taken immunosuppression as its core feature, as it plays a decisive role in the production and proliferation of tumor cells [4]. Tumor microenvironment is a unique environment that involves interactions of tumor cells, including tumor stroma, adjacent cells, various immune cells around blood vessels and other immune-related mediators, with the host. Changes in various components of tumor microenvironment demonstrated important effects on tumor growth, invasion, metastasis and tumor immune tolerance [5]. The occurrence and development of STAD is based on the coordinated evolution of cancer cells and tumor immune microenvironment. Surgery, radiotherapy, and chemotherapy can aggravate immunosuppression, promoting the proliferation of residual cancer cells or the recurrence of tumor cells [6]. Therefore, to improve immune surveillance, restrain immunosuppression and either activate or inhibit immune escape, it is imperative to improve the immune microenvironment of gastric cancer, affecting the growth, progression and outcome of gastric cancer. This becomes a new target for the prevention and treatment of gastric cancer [7]. Immunization and stromal cells are the two major non-tumor components in the tumor microenvironment and has great value in the diagnosis and prognosis assessment of tumors [8]. Yoshihara et al. have designed an algorithm called ESTIMATE, which is used for calculating immune and stromal scores for predicting the infiltration of non-tumor cells using gene expression data from the The Cancer Genome Atlas (TCGA) database [8].

In this study, the immune and stromal scores of patients with STAD were calculated, and the scores in STAD with different clinicopathological features from the TCGA datasets were systematically compared. Next, a list of genes that are correlated with microenvironment was extracted, and predicted the prognosis and survival in STAD patients via TCGA database of STAD cohorts and ESTIMATE algorithm-derived immune scores. Glycoprotein non-metastatic melanoma protein B (GPNMB) was selected from the hub genes based on survival and prognosis analyses. Multivariate cox regression analyses were performed and a nomogram was built with potential risk factors based on a multivariate Cox analysis to predict the survival probability. Furthermore, cell function experiments and xenograft tumors *in vivo* were performed to verify the role of it in tumor progression.

## RESULTS

### Association of immune scores and stromal scores with STAD

**Figure 1:**
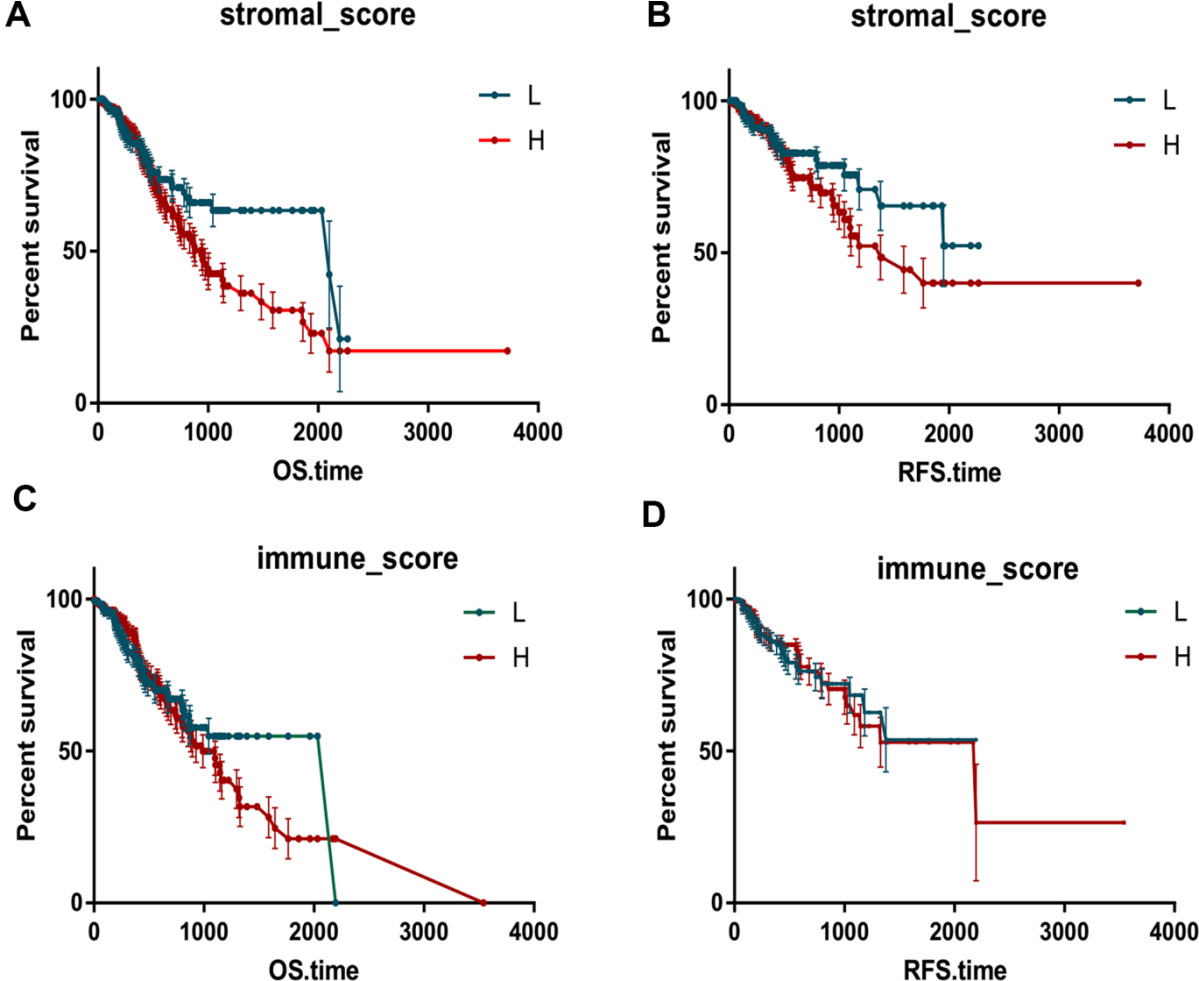
**Immune scores and stromal scores are associated with Kaplan-Meier survival in STAD.** The OS and RFS curves between high and low groups based on immune scores (**A**, **B**). The OS and RFS curves between high and low groups based on immune scores (**C**, **D**).

**Figure 2:**
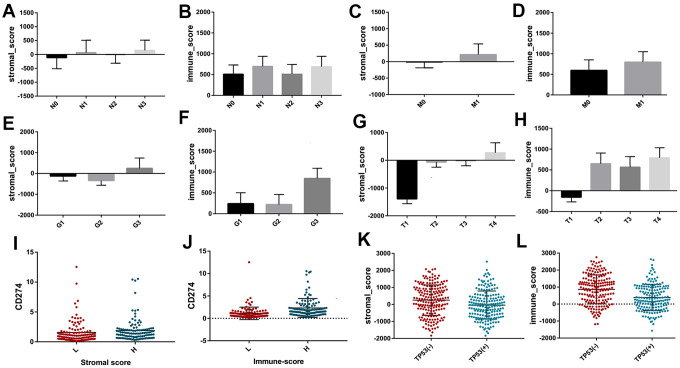
The different distribution of stromal scores and immune scores in different clinicopathological features, including stage N (**A**, **B**) stage M (**C**, **D**) grade (**E**, **F**) and stage T (**G**, **H**). The different expression of CD274 between high and low groups based on immune scores and stromal scores (**I**, **J**). The different distribution of stromal scores and immune scores between different TP53 mutation and wildtype (**K**, **L**).

To investigate the prognostic value of immune-related scores (stromal scores and immune scores) in STAD, all the 317 STAD cases were divided into high and low groups based on their immune and stromal scores to find the association between the survival rate and the scores. The results revealed that patients with low stromal scores demonstrated slightly increased overall and disease-free survival rates when compared to high stromal scores (Figure 1, P < 0.05), while the overall survival rate of low immune scores was much higher than that of high immune scores (Figure 1, P < 0.05). Next, the association between the scores and clinical information were analyzed based on the data obtained from the TCGA database. Among them, the stromal scores and immune scores showed positive correlation with stages T and M as well as grade, indicating that the immune-related scores might contribute to STAD progression, while only stromal scores were positively correlated with stage N (Figure 2, P < 0.005). In addition, the immune-related scores were analyzed according to CD274 expression and TP53 mutation states in STAD. As shown in Figure 2, TP53 mutant cases had lower scores when compared to those with wild-type TP53 and CD274 expressed in high score group are higher than that of low group.

### Identification and analysis of DEGs and Hub genes

**Figure 3:**
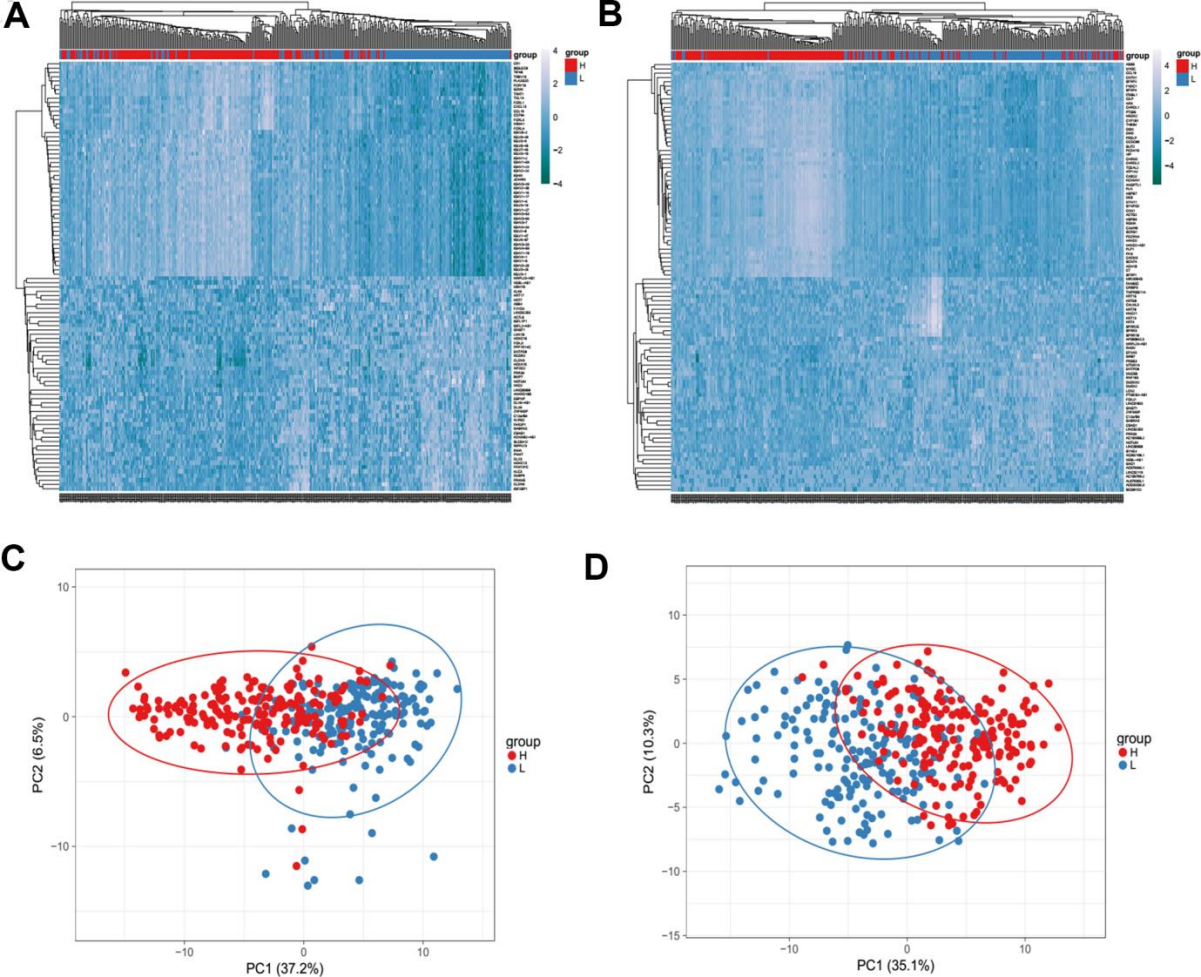
Heatmaps (**A**, **B**) and Principal Component Analysis (PCA) (**C**, **D**) showed different gene expression profiles in the immune scores of top half (high score) vs. bottom half (low score). P <0.05, fold change >1.5. Genes with higher expression are shown in red, lower expression are shown in blue**.**

**Figure 4:**
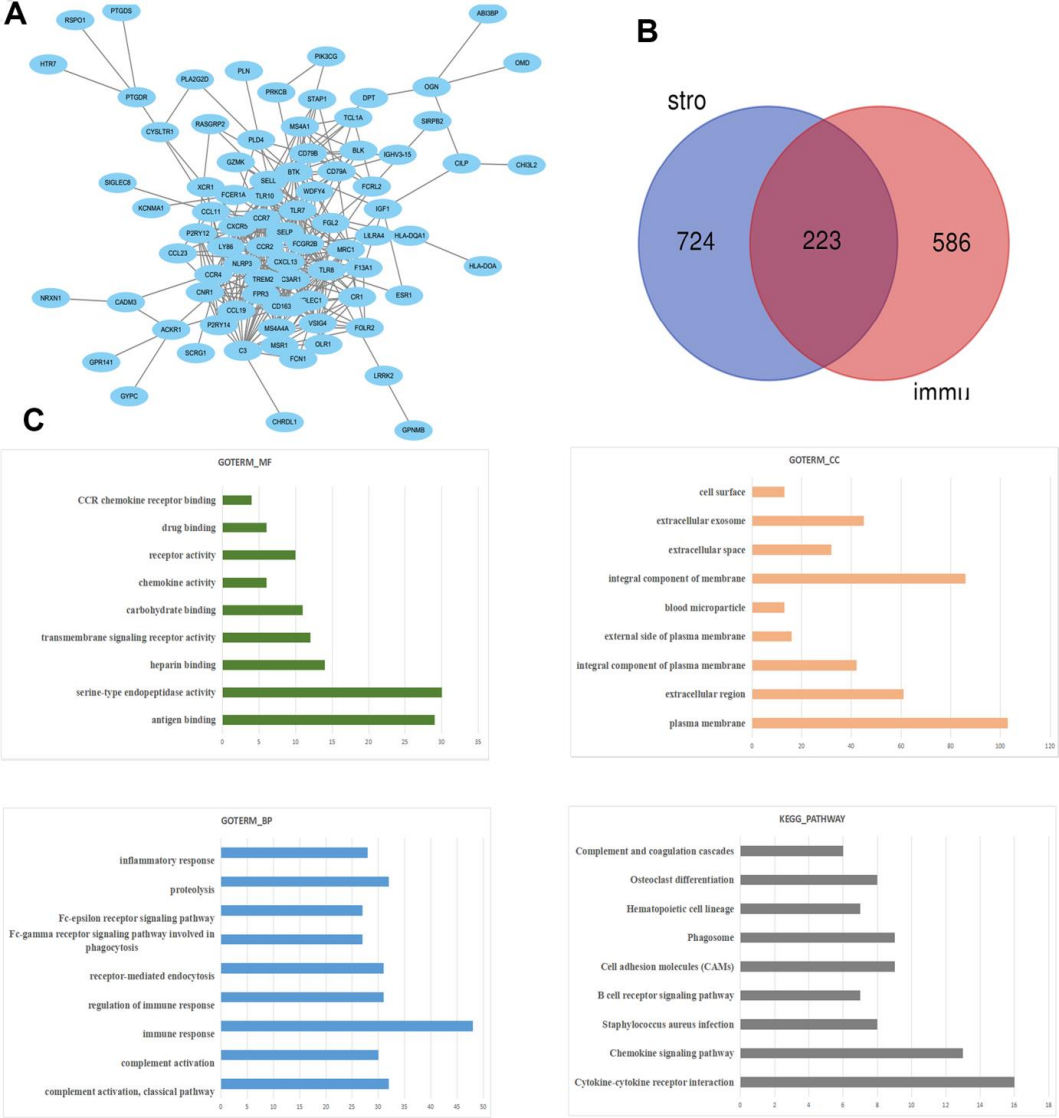
**The PPI network of DEGs was constructed using Cytoscape.** (**A**) Venn diagram was generated on the stromal scores and immunes scores of DEGs via Draw Venn Diagram (**B**). The biological process analysis of hub genes was constructed using BiNGO. The color depth of nodes refers to the corrected P-value of ontologies. The size of nodes refers to the numbers of genes that are involved in the ontologies. P<0.05 was considered statistically significant. The KEGG pathway and GO enrichment analysis of DEGs (**C**).

Next, the Agilent G microarray data of all 510 STAD cases obtained from the TCGA database was examined to find out the association of gene expression profiles. Heatmaps showed different gene expression profiles in the high immune-related scores vs low immune-related scores group (Figure 3A, 3B). Next, 223 DEGs were selected based on immune scores and stromal scores (fold change >1.0, P < 0.05), (Figure 4B). Also PCA and PPI network were constructed using the ClustVis (https://biit.cs.ut.ee/clustvis/), (Figure 3C, 3D and Figure 4A).

The KEGG pathway and GO enrichment analysis of DEGs were performed by DAVID (Figure 4C). The results of GO analysis revealed that the biological processes of DEGs showed significant enrichment in immune response, proteolysis, inflammatory response b and regulation of immune response. Molecular functions of DEGs demonstrated advancement in antigen binding, serine-type endopeptidase activity and transmembrane signaling receptor activity. The cell components that are enriched with DEGs included plasma membrane, integral component of membrane and extracellular region. KEGG pathway analysis revealed that DEGs were mainly enriched during cytokine-cytokine receptor interaction, chemokine signaling pathway, Staphylococcus aureus infection, and B cell receptor signaling pathway.

**Figure 5:**
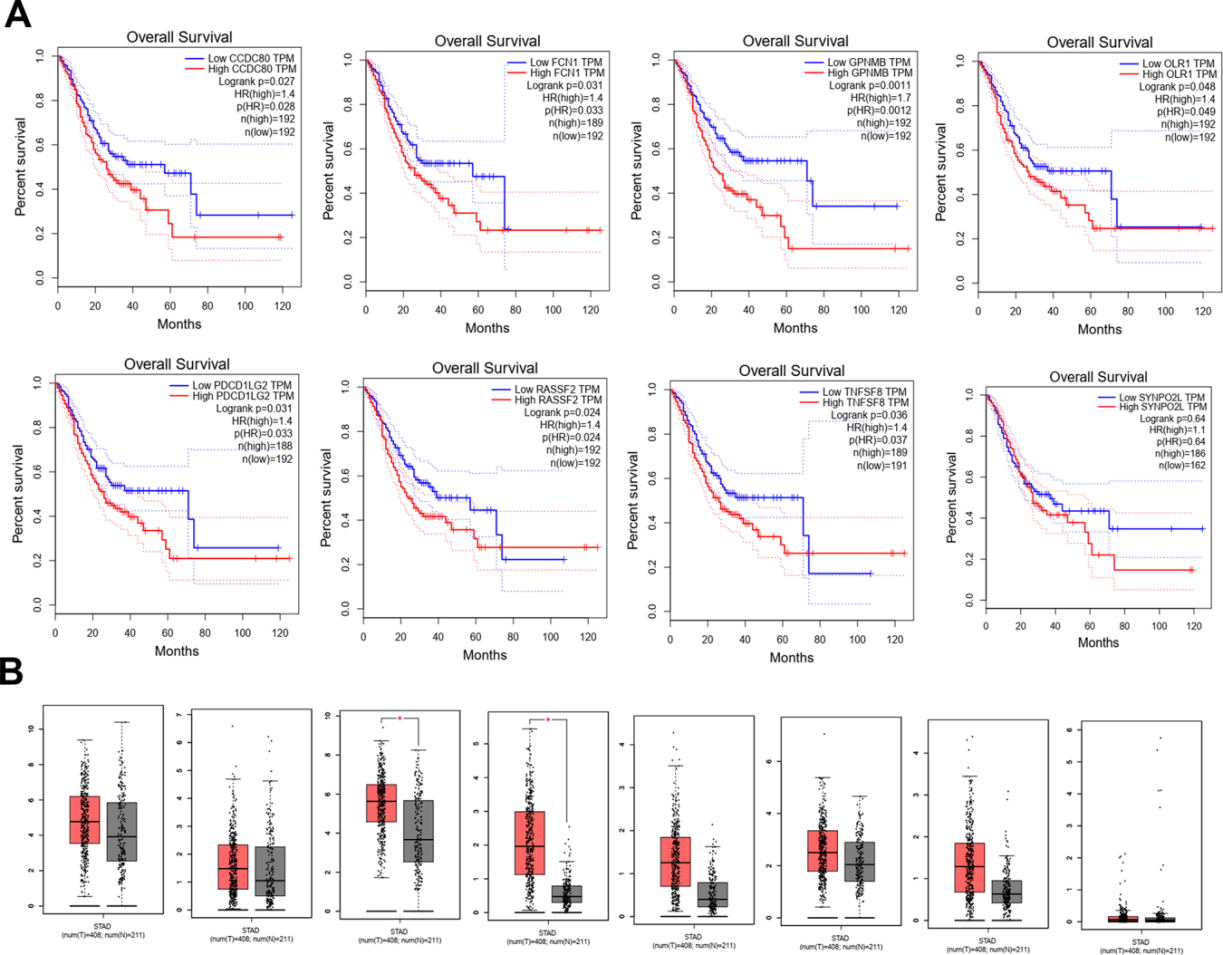
**Kaplan-Meier plots the 8 hub genes by R v3.5.1 from TCGA database.** (**A**) The different expression levels of 8 hub genes expression between normal and tumor from TCGA (**B**).

Eight hub genes were selected using BiNGO and were presented in Table 1. The selection criteria were as follows: MCODE scores >5, degree cut-off=2, node score cut-off=0.2, Max depth=100 and k-score=2. Next, Kaplan-Meier plots of the 8 hub genes were upregulated in the patients, decreasing the overall survival rate (Figure 5A). Interestingly, compared to normal tissues, GPNMB and TNFSF8 were significantly over-expressed in patients with STAD (P < 0.05) (Figure 5B).

**Table 1:** Functional roles of 8 hub genes with degree ≥10.

**Gene symble**	**Full name**	**Function**
CCDC80	Coiled-Coil Domain Containing 80	Diseases associated with CCDC80 include Herpes Zoster Oticus and Peripheral Nervous System Disease. Gene Ontology (GO) annotations related to this gene include heparin binding and glycosaminoglycan binding..
FCN1	Ficolin 1	Among its related pathways are Innate Immune System and Creation of C4 and C2 activators. Gene Ontology (GO) annotations related to this gene include calcium ion binding and antigen binding.
GPNMB	Glycoprotein Nmb	Among its related pathways are Adhesion and Signaling by GPCR. Gene Ontology (GO) annotations related to this gene include heparin binding and integrin binding. An important paralog of this gene is PMEL.
OLR1	Oxidized Low Density Lipoprotein Receptor 1	Among its related pathways are Innate Immune System and Cell surface interactions at the vascular wall. Gene Ontology (GO) annotations related to this gene include carbohydrate binding and low-density lipoprotein particle receptor activity.
PDCD1LG2	Programmed Cell Death 1 Ligand 2	Involved in the costimulatory signal, essential for T-cell proliferation and IFNG production in a PDCD1-independent manner. Interaction with PDCD1 inhibits T-cell proliferation by blocking cell cycle progression and cytokine production.
RESSF2	Ras Association Domain Family Member 2	RASSF2 (Ras Association Domain Family Member 2) is a Protein Coding gene. Among its related pathways are Hippo signaling pathway - multiple species. An important paralog of this gene is RASSF4.
TNFSF8	TNF Superfamily Member 87	Diseases associated with TNFSF8 include Anaplastic Large Cell Lymphoma and Lymphoma, Hodgkin, Classic. Among its related pathways are ERK Signaling and Akt Signaling. Gene Ontology (GO) annotations related to this gene include signaling receptor binding and tumor necrosis factor receptor binding.
C2	Synaptopodin 2 Like	Actin-associated protein that may play a role in modulating actin-based shape.

### Prognosis of GPNMB in STAD patients

**Figure 6:**
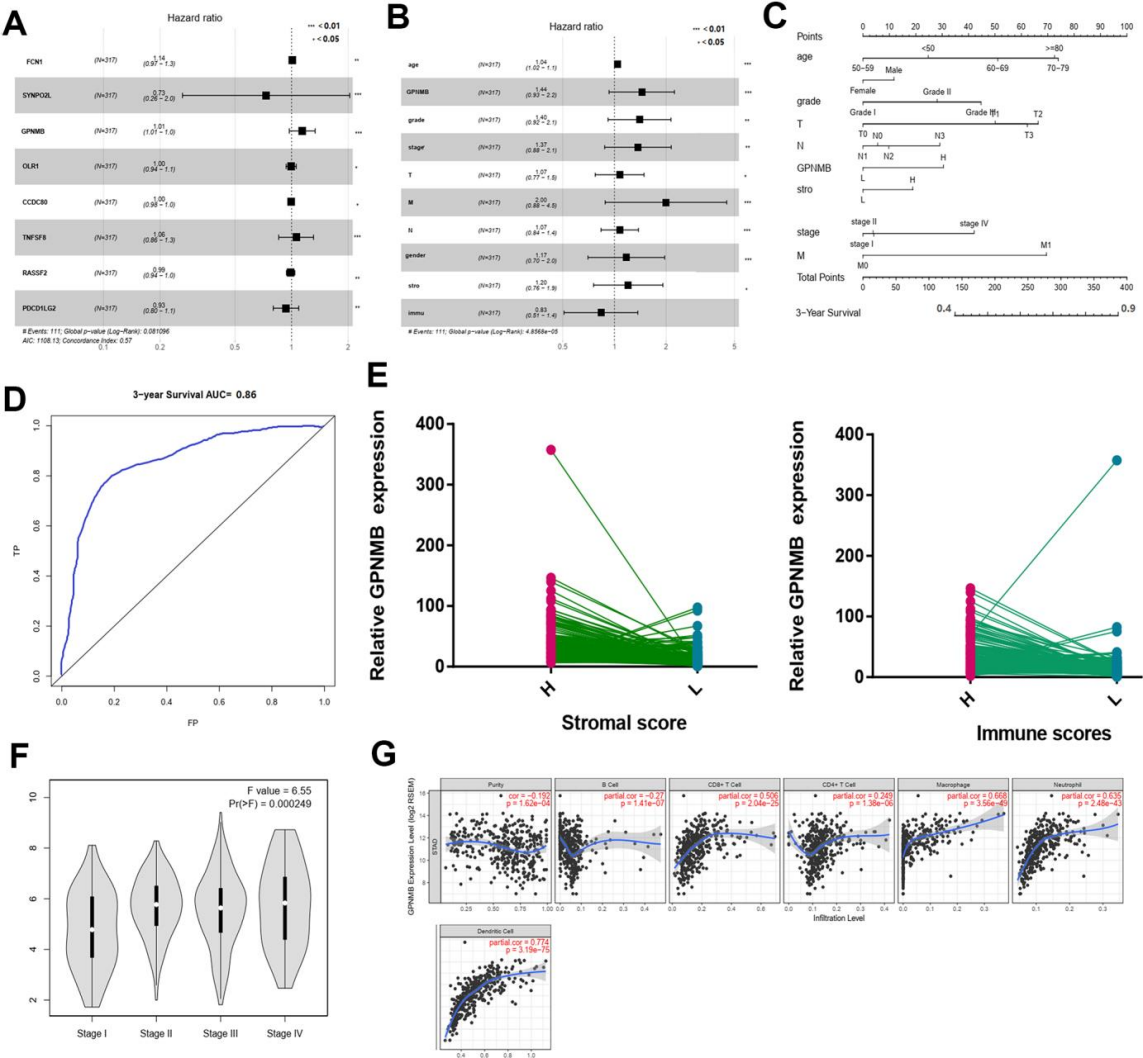
**Multivariate Cox regression analyses among the hub genes.** (**A**) and related clinical characteristics respectively (**B**). The prognostic nomogram that integrated all significant independent factors from the multivariate analysis for OS in the training cohort (**C**). The RCO curve area evaluating the prognostic nomogram for OS prediction was 0.860 (**D**). The relate GPNMB expression between high and low immune scores (**E**). The different expression of GPNMB in STAD with different stage features (**F**). The correlation of GPNMB expression with immune infiltration level in STAD via TIMER (**G**).

A total of 317 STAD patients were obtained from the TCGA. The demographic and clinicopathological characteristics of patients are listed in Table 2. The results of multivariate analysis of the 8 genes are listed in Figure 6A. Among these genes, GPNMB was considered as significantly risky gene with HR > 1 and P < 0.05. Multivariate analysis of GPNMB expression, related scores and clinicopathological characteristics revealed that its expression, grade, stage and stromal scores were statistically significant factors for the progression of STAD (Figure 6B). In addition, all the input factors should be incorporated into the nomogram later as adjustment items. The prognostic nomogram by integrating all significant independent factors from the multivariate analysis for predicting the overall survival rate in the training cohort was shown in Figure 6C. The area under the curve of ROC in prognostic nomogram for overall survival prediction was 0.86 (95%CI, 0.80 to 0.92) (Figure 6D). Interestingly, compared to low scores group, GPNMB showed significant overexpression in high immune-related score STAD group (Figure 6E). Also expression of GPNMB showed positive correlation with stage and immune infiltration including CD8+T, macrophages, neutrophils and dendritic cells (Figure 6F, 6G).

**Table 2:** Demographic and clinicopathological characteristics of patients with STAD in TCGA database.

**Demographic or Characteristics**	**All subjects (N=317)(%)**	**Training cohort (N=167)**	**Validation cohort (N=150)**
**Age at diagnosis**			
<=49	27(8.5)	13(8.0)	12(8.0)
50-59	82(25.9)	43 (26.0)	39(25.8)
60-69	109(34.4)	58(34.5)	51(34.4)
70-79	105(33.1)	56(33.5)	49(33.0)
>=80	22 (6.9)	12(7.0)	10(6.9)
**Gender**			
Female	120(37.9)	63(37.8)	57(38.8)
Male	197(62.1)	104(62.2)	93(61.2)
**Stage**			
I	42(13.2)	23(13.5)	19(13.2)
II	101(31.9)	53(32.0)	48(32.3)
III	139(43.8)	73(44.0)	66(43.6)
IV	35(11.0)	18(10.5)	17(9.9)
**Grade**			
Grade I	7(2.2)	3(2.0)	4(2.1)
Grade II	108(34.1)	57(34.0)	51(33.0)
Grade III	202(63.7)	107(64.0)	95(63.0)
**T**			
T1	15(4.7)	8(4.7)	7(4.4)
T2	63(19.9)	34(20.0)	29(20.6)
T3	152(47.9)	82(49.0)	85(47.8)
T4	87(27.4)	47(28.0)	40(27.5)
**M**			
M0	295(93.0)	152(91.0)	143(94.0)
M1	22(7.0)	12(7.5)	10(6.9)
**N**			
N0	70(22.1)	37(22.0)	33(22.2)
N1	83(26.2)	45(27.0)	38(26.8)
N2	69(21.8)	35(21.0)	34(20.2)
N3	66(20.8)	35(21.0)	31(20.8)
**GPNMB expression**			
High	160(50.5)	85(51.0)	75(50.0)
Low	157(49.5)	82(49.0)	75(50. 0)
**Stromal scores**			
High	158(49.8)	84(50.0)	74(50.0)
Low	159(49.2)	83(50.0)	76(50.0)
**Immune scores**			
High	160(50.0)	84(50.0)	74(50.0)
Low	157(50.0)	83(50.0)	76(50.0)

### GPNMB promotes proliferation of STAD cell lines

**Figure 7:**
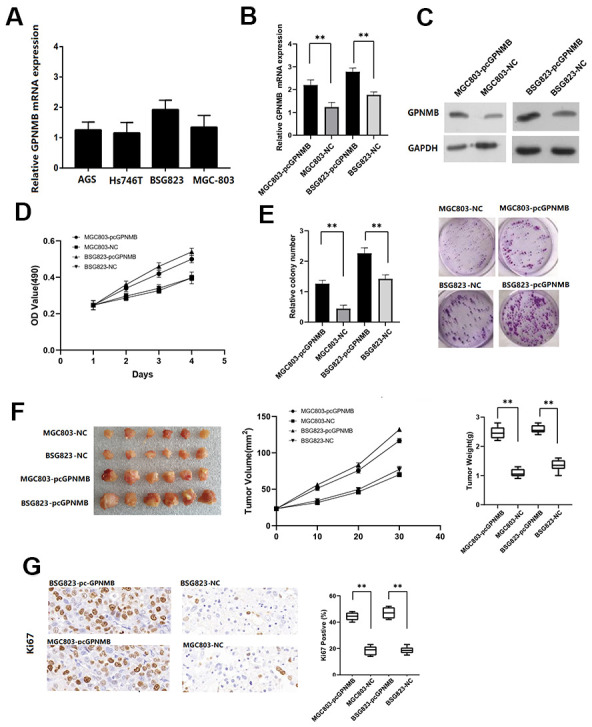
**The GPNMB expression in different STAD cells.** (**A**) The expression efficiency of pc-GPNMB was measured by RT-Qpcr (**B**) and WB assay (**C**) in MGC803 and BSG823 cells. MTT assays show that GPNMB overexpression increased cell proliferation in MGC803 and BSG823 cells (**D**). Colony formation assays indicate significantly increased the number of colonies in pc-GPNMB group (MGC803 and BSG823 cells) cells compared to NC group (**E**). The plots showing tumor growth measurements of MGC803-pc-GPNMB, MGC803 (control), BSG823- pc-GPNMB and BSG823 (control) cells is shown, and the mean tumor weights in each group (MGC803-pc-GPNMB, MGC803, BSG823-pc-GPNMB and BSG823) on day 30 is shown (**F**). IHC staining showing that cell proliferation (Ki67-positive) positively correlates with GPNMB expression levels (**G**). All the above experiments were repeated six times respectively. *P < 0.05 versus control.

Firstly, RT-qPCR assay was performed to investigate the expression of GPNMB in different STAD cell lines. The results showed that GPNMB expression was markedly upregulated in MGC803 and BSG823 cell lines (Figure 7A). The expression efficiency of pc-GPNMB was measured by RT-qPCR assay and western blotting in both MGC803 and BSG823 cell lines. Next, GPNMB was overexpressed by transfecting GPNMB plasmid into MGC803 and BSG823 cell lines, indicating that transfection of pc-GPNMB was effective and can be used for subsequent research (Figure 7B, 7C). The effects of GPNMB upregulation on cell proliferation and apoptosis were further examined in MGC803 and BSG823 cell lines. MTS analysis showed that the proliferation ability of MGC803 and BSG823 cells with GPNMB overexpression was significantly higher than that of the mock cells (Figure 7D). Colony formation analysis showed that GPNMB overexpression caused a significant increase in the number of colonies in MGC803 and BSG823 cell lines (Figure 7E).

### GPNMB promotes STAD growth *in vivo*

To further study the biological significance of GPNMB in STAD patients, MGC803-pcGPNMB and BSG823-pcGPNMB cells were subcutaneously injected and their corresponding controls into nude mice and monitored their tumor growth. Compared to the control cells, MGC803-pcGPNMB and BSG823-pcGPNMB cells demonstrated a significantly larger tumor size. In addition, the high GPNMB levels in STAD cells showed association with higher tumor growth rates and larger tumor weight (Figure 7F). Immunohistochemical experiments revealed that the Ki67 positive rate in high-GPNMB group was significantly higher than that of the control group (Figure 7G). Taken together, these data suggest that GPNMB promotes the growth of STAD tumors *in vivo*.

### mir-30b-3p targets GPNMB

**Figure 8:**
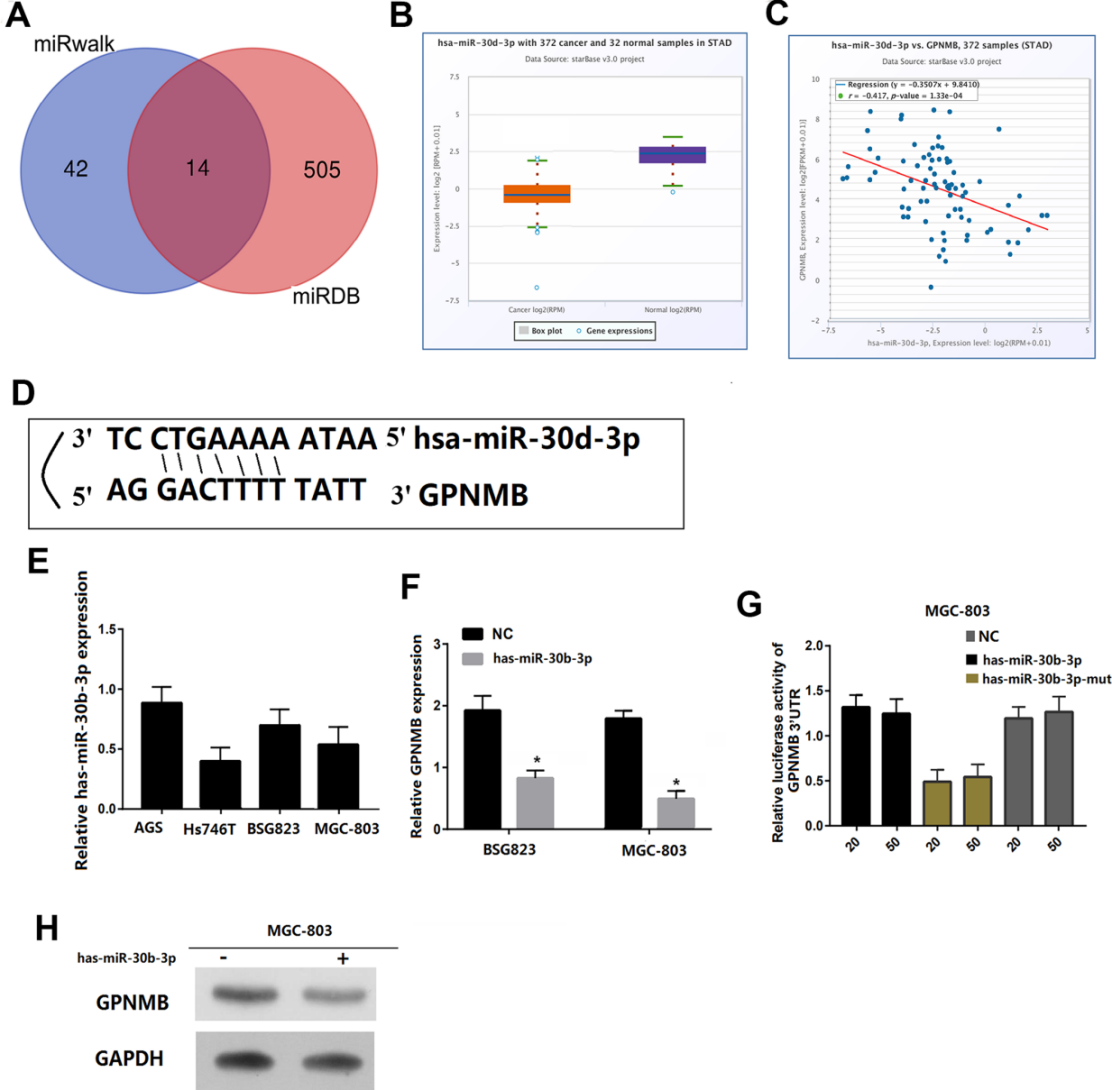
**GPNMB is a target for miR-30b-3p in STAD cells. Venn diagrams showing the number of potential miRNAs targeting the 3’UTR of GPNMB, as predicted by two databases, miRanda and miRDB.** (**A**) The gene expression of miR-30b-3p between cancer and normal samples via ENCORI. (**B**) The coexpression between miR-30b-3p and GPNMB via ENCORI showed that miR-30b-3p expression is negatively correlated with GPNMB expression. (**C**) Sequences of miR-30b-3p and their potential binding sites in the 3’UTR of GPNMB is shown. (**D**) Quantitative real time PCR analyzing miR-30b-3p expression relative to MGC803 as internal control is shown. (**E**) Comparison of GPNMB expression in STAD cells transfected with miR-30b-3p mimic or negative control (NC) based on qRT-PCR (**F**), and western blotting (**H**), and the loading control for western blotting was GADPH. Analysis of luciferase activity from reporters containing the 3’UTR end of GPNMB in cells transfected with the miR-30b-3p mimic, miR-30b-3p mutation mimic (miR-30b-3p-mut) and negative control (NC) is shown. (**G**) All the above experiments were repeated six times respectively. *P < 0.05 versus control.

Finally, two sets of miRNAs related to GPNMB were selected from miRanda and miRDB databases and obtained miR-30p-3b as a possible target candidate (Figure 8A). The expression of miR-30p-3b between tumors and normal tissues was compared via ENCORI. The results showed that the expression of miR-30p-3b in tumors was lower than that of normal tissues (P < 0.05) (Figure 8B). miRNA-target coexpression between miR-30p-3b and GPNMB via ENCORI showed that miR-30p-3b expression was negatively correlated with GPNMB expression (Figure 8C). More importantly, miR-30p-3b might be combined with 3’ UTR of GPNMB (Figure 8D). Therefore, miR-30p-3b might be speculated to act as a potential key upstream negative regulator of GPNMB and might be related to cancer treatment. The expression levels of GPNMB between miR-30p-3b and control groups in MGC803 and MSG823 cell lines was evaluated by qRT-PCR, and the results showed that GPNMB was down-regulated in both cells (Figure 8E, 8F). To determine the role of miR-30p-3b in regulating GPNMB expression, MGC803 cells or mutant miR-30p-3b mimics were transfected to luciferase-labeled NEK2-3'UTR, and then were analyzed by luciferase reporter gene analysis. The results showed that miR-30p-3b mimic has significantly reduced the luciferase activity of GPNMB-3'UTR, while the mutant miR-30p-3b mimic did not inhibit the luciferase activity of GPNMB -3'UTR (Figure 8G). Furthermore, the expression levels of GPNMB in MGC803 cells transfected with miR-30p-3b or negative control (NC) were analyzed by qRT-PCR and western blotting, and the results showed that the expression of GPNMB with miR-30p-3b was significantly lower than that of negative control group (Figure 8H). Therefore, these results indicated that GPNMB was upregulated by miR-30p-3b and might have an impact on the prognosis of STAD patients.

## DISCUSSION

Increasing evidence has shown that the differences in the effectiveness of tumor immunotherapy can be attributed to inhomogeneities of tumor microenvironment [9, 10]. The tumor microenvironment is composed of tumor cells, tumor infiltrating immune cells and extracellular matrix (ECM), which together promote tumor growth, transformation and invasion, protect tumor cells from immune surveillance by the host, and cause tumors to develop resistance and provide the basis for tumor dormancy and metastasis [11]. Recent studies have revealed that immune microenvironment directly or indirectly affects tumorigenesis and development. The mechanism involves promotion of tumor angiogenesis, changing of tumor biological characteristics, screening of tumor cells that are adapted to the microenvironment for survival, and promotion of tumor progression by establishing a suitable tumor microenvironment [12]. In this study, we demonstrated that patients with low stromal scores had slightly increased overall survival and disease-free survival rates when compared to that of high stromal scores, while the overall survival rate of low immune scores was much higher than that of high immune scores. The stromal scores and immune scores were positively correlated with stages T and M and grade, indicating that the related immune scores might contribute to STAD progression (Figure 1B, P < 0.005).

Sun et al. have measured the expression levels of PD-L1 in cancer tissues and normal tissues of 102 patients with gastric cancer, and the results showed that the positive expression rate of PD-L1 in gastric cancer tissues was 40%-63%, while hardly detected in normal gastric tissues [13]. Zhang et al. have conducted meta-analysis of 10 clinical studies including 1901 patients with gastric cancer, and found that the positive expression rate of PD-L1 in gastric cancer was significantly higher than that in normal gastric tissues, yielding better immunotherapy effects [14]. In this study, the expression of PD-L1 with different immune scores and stromal scores revealed that the expression of PD-L1 in high score group was higher than that of low score group. Also the high expression of PD-L1 might induce apoptosis of anti-tumor T lymphocytes, and achieve immune escape of gastric cancer cells, promoting the occurrence and development of gastric cancer. Many studies have shown that TP53 mutations play a negative role in antitumor immunity [15, 16]. Nilay revealed that deletion of TP53 in gastric cells conferred a selective advantage and promoted the development of dysplasia in the setting of dietary carcinogenesis [17]. TP53 mutations frequently occur in STAD patients and are associated with unfavorable clinical outcomes. To explain these findings, the immune scores and stromal scores of gastric cancer cohorts between TP53 mutant state from TCGA project were analyzed. The results showed that TP53 mutant cases had lower immune and stromal scores when compared to those with wild-type TP53. TP53 mutation itself results in decreasing the immune activities in STAD and other cancer types, and immune cell infiltration and immune activities showed a positive association with survival prognosis in STAD [18].

Next, 223 DEGs were selected based on the immune scores and stromal scores. The KEGG pathway and GO analysis showed significant enrichment of DEGs during immune response and its regulation. The 8 hub genes were selected using BiNGO. Kaplan-Meier plots of the 8 hub genes were upregulated in STAD patients, decreasing their overall survival. Interestingly, compared to normal tissues, GPNMB was significantly over-expressed in STAD (P < 0.05). GPNMB was discovered in 1995 by Weterman et al. [19], which is a type I transmembrane glycoprotein, and forms a new signal transduction pathway with Melanocyte Inducing Transcription Factor (MITF), and might promote the development of human cancer. Some reports have suggested that GPNMB might be involved in the differentiation of tissue cells and metastasis of tumor cells [20, 21], and is associated with the occurrence and invasion of melanoma cells, glioma cells, breast [22–24], colorectal cancer [25], and prostate cancer [26] cells. Tomihari confirmed that GPNMB in patients with melanoma has the ability to downregulate the activation of melanoma-reactive T cells, thereby allowing melanoma to evade immunologic recognition and destruction [27]. Identification of biomarkers that distinguish responders and nonresponders might improve the management of patients with cancer. GPNMB checkpoint differs from that of the PD1 signaling pathway in expression and inhibitory mechanisms, and GPNMB expression might regulate immune checkpoint inhibitors responsiveness [28]. These studies were consistent with the findings of our study with regard to the association of high expression of GPNMB in STAD with worse prognosis and advanced progression. Interestingly, we found that the expression of GPNMB was positively correlated with immune infiltration cells including CD8+T, macrophages, neutrophils and dendritic cells. In addition, multivariate analysis revealed that age, grade, stage, stromal scores and GPNMB were considered as statistically significant factors for STAD progression. A prognostic nomogram has integrated all significant independent factors obtained from the multivariate analysis for overall survival. Wang et al. have developed a significant prognostic nomogram for predicting the respective overall survival rates of STAD, and the C-Index for overall survival prediction was 0.707 [29]. However, our nomogram has 8 readily available pathological variables and achieved a C-index of 0.860, which was superior to that of other nomograms and larger than that of the traditional seventh AJCC staging system (0.860vs 0.661). This indicated that high expression of GPNMB acts as an independent risk factor and improved the current prognostic model. Furthermore, cell function experiments and xenograft tumors *in vivo* were performed to further verify the role of GPNMB in tumor progression, and the data analysis results were presented above.

MicroRNAs (miRNAs) are a wide range of regulatory non-coding RNAs (18-25 nt) in mammals and can either activate or inhibit the expression of target genes through post-transcriptional regulation. At present, the abnormally expressed miRNAs are always used as key factors in disease prediction, prevention and treatment [30–32]. Based on the miRanda and miRDB databases, miR-30b-3p might upregulate and bind to 3’UTR of GPNMB, which was confirmed by fluorescent enzyme reporter gene experiment. MiR-30b-3p is either upregulated or downregulated in several types of cancers. Previous studies have revealed that miR-30b-3p is downregulated in primary prostate cancer (PCa) and metastatic castration resistant PCa and can directly inhibit androgen receptor and PCa cell proliferation [33]. The expression of miR-30b-3p is markedly decreased in hepatocellular cancer tissues and cells, showing positive correlation with higher overall survival rate [34]. The expression of miR-30p-3b in tumors was higher than that of normal tissues (P < 0.05). More importantly, miR-30p-3b might be combined with 3 ’UTR of GPNMB, and miR-30p-3b expression was negatively correlated with GPNMB expression, which was confirmed by fluorescent enzyme reporter gene experiment. Therefore, we speculated that hsa-miR-346 acts as a potential key upstream negative regulator of GPNMB and might assist in treating cancer.

In summary, the prognostic value of stromal scores and immune scores in STAD was confirmed, and the hub genes were selected and analyzed from DEGs. Importantly, GPNMB was frequently overexpressed in STAD and was associated with aggressive STAD, and high GPNMB expression also indicated poor prognosis and progression in patients with STAD. Next, a nomogram was developed to predict the overall survival of patients with STAD. Finally, miR-30b-3p was identified as an upstream regulator of GPFMB expression, which might be developed as a new treatment strategy in reducing the development of STAD.

## MATERIALS AND METHODS

### Microarray data

The gene expression profiles and clinical information were downloaded from TCGA database, which is a landmark in cancer genomics program, and involves molecular characterization of over 20,000 primary cancer and matched normal samples spanning 33 cancer types. The stromal scores and immunes scores were calculated by an open source web tool ESTIMATE (https://bioinformatics.mdanderson.org/estimate/). Kaplan-Meier plots were then constructed to analyze the overall survival and disease-free of the stromal scores and immunes scores. Next, the stromal scores and immune scores in STAD with different clinicopathological features, including stage T/N/M, CD274 expression and TP53 mutation, were systematically compared.

### Identification and analysis of DEGs

Heatmaps and principal component analysis (PCA) were constructed using R software. Differentially expressed genes (DEGs) were then identified using the limma package of the R statistical software (Fold change > 1.5 and adjusted P < 0.05 were set as the cutoff values). Venn diagram was generated based on the stromal scores and immune scores of DEGs via Draw Venn Diagram (http://bioinformatics.psb.ugent.be/webtools/Venn/). The protein-protein interaction (PPI) network was made using The Search Tool for the Retrieval of Interacting Genes (STRING). The KEGG pathway and GO enrichment analysis of DEGs were performed by DAVID (http://david.ncifcrf.gov). The top signaling pathways with false discovery rate (FDR) of <0.05, and -log FDR >1.5 were identified as significant cut-off values.

### Hub genes analysis and survival curve

The hub genes were selected using BiNGO (Cytoscape). Kaplan-Meier plots were constructed to analyze the overall survival and disease-free survival rates of the 5 hub genes by R v3.5.1. The expression levels of hub genes between normal and turmor from the TCGA database were compared using the R software.

### Prognostic nomogram

Multivariate Cox regression analyses were performed to determine the prognostic value of the hub genes and related clinical characteristics. The nomogram was built with potential risk factors (P <0.05) based on multivariate Cox analysis using the R software package. The predictive performance of the nomogram was measured by operating characteristic (ROC) curves. The relation between high and low scores of GPNMB expression was performed, and also the expression of GPNMB in STAD with different stage features was compared. The correlation of its expression with immune infiltration level in STAD was performed via TIMER (Tumor IMmune Estimation Resource), which is a web server used for comprehensive analysis of tumor-infiltrating immune cells (https://cistrome.shinyapps.io/timer/).

### Cell culture and transfection

STAD cell lines were cultured in DMEM medium containing 10% fetal bovine serum under the conditions of 37C, 5% CO2, and 100% saturated humidity. Passive culture was performed when the cells confluent to 70% to 80%. The cells were cultured in 6-well plates and synchronized with serum-free medium for 24 hours. Further, the cells were divided into pc-NC groups and pc-GPNMB groups. Next, we transfected the blank plasmid and GPNMB-interfering plasmid following the instructions of the Lipofectamine 2000 transfection kit.

### Quantitative real-time polymerase chain reaction and Western blotting

Qqt-PCR was used to detect the changes in the GPNMB expression. The aforementioned reverse transcription products were tested by Takara's SYBRPremixExTaqTM on an ABI7900 instrument using qPCR, and GAPDH expression was utilized as an internal reference. The following primers were used: GPNMB - TGCCAAGCGATTTCGTGATGT; GAPDH - ACCCACTCCTCCACCTTTGA, CTGTTGCTGTAGCCAAATTCGT. Cells from pc-NC group and pc-GPNMB group cells were collected, and total cell protein was extracted using cell lysate. Then the protein concentration was determined by a BCA kit. According to the results of protein concentration detection, 25uL of protein samples were subjected to Western blot detection.

### Colony formation assay

Transfected MGC803 and BSG823 cells were seeded into 60 mm culture dishes and cultured for 14 days in the complete medium. Then, cells were fixed using methanol for 10 min and stained with 0.1% crystal violet (Sigma-Aldrich) for 15 min. Next, dishes were photographed and colonies with over 50 cells were counted.

### *In vivo* xenograft tumor growth

Twelve nude mice were divided into an experimental and a control group (n = 6). Nude mice were kept in an environment of about 25 °C with a suitable humidity, and were provided with sufficient food and water during the administration. Experimental grouping: 5 × 106 cells transfected with pc-GPNMB in MGC803 cell (pc-GPNMB in BSG823 cell) were suspended in 0.1 mL of PBS and injected subcutaneously into nude mice. The control group was given the same amount of normal saline. The volume and weight of the xenograft tumors in the nude mice were then measured every ten days. Thirty days later, the nude mice were euthanized, and the transplanted tumors were removed. All animal experiments were performed as approved by the Committee of Huaihe hospital of Henan university.

### Immunohistochemical analysis

Antibody system immunohistochemistry kits were purchased from Roche, Switzerland. According to the method of Dowset et al. [35], pale yellow, yellow or brown particles appeared in the nuclei of cells as positive expression. Readers blinded to the patient's pathological data, observed the expression of the whole film under a microscope. The expression levels of GPNMB protein were divided into control group and high expression groups.

### miRNA database analysis

The potential miRNAs targeting GPNMB were downloaded from miRanda and miRDB databases. We performed survival analysis of miR-30d-3p in STAD downloaded from TCGA. The expression values of miRNAs from miRNA-seq data were scaled with log2(RPM+0.01). Co-expression analysis for miR-30d-3p and GPNMB were performed via ENCORI, which mainly focuses on miRNA-target interactions and is an open-source platform for studying the miRNA-ncRNA, miRNA-mRNA and RBP-mRNA interactions from CLIP-seq, degradome-seq and RNA-RNA interactome data (https://web.archive.org/web/20110222111721/http://starbase.sysu.edu.cn/).

### Luciferase reporter assay

The GPNMB gene promoter was cloned by RT-q PCR and DNA fragments from the 3’-UTR of GPNMB inserted into the luciferase reporter vector pGL3. The STAD cells (10,000/well) were seeded in triplicate in 48 well plates. Then, the pGL3- GPNMB -3’UTR reporter plasmids (100 ng) plus 5 ng of pRL-TK renilla plasmid and increasing levels of negative control (NC), miR-30d-3p or mutant miR-30d-3p mimics were co-transfected into the MGC803 cells. Luciferase activity analysis was next performed to calculate the luciferase activity ratio of the reporter plasmid and the internal reference.

### Statistical analysis

Statistical analysis was performed with SPSS software and R software (version 3.5.1). Factors were identified as significant at (PP < 0.1) in the univariate analysis. Comparisons between groups were performed using independent sample t-tests, and multiple group comparisons were performed using single-factor variance, and pairwise comparisons were performed using LSD Lt test. PP < 0.05 was considered statistically significant.
